# Repair of critical sized cranial defects with BMP9-transduced calvarial cells delivered in a thermoresponsive scaffold

**DOI:** 10.1371/journal.pone.0172327

**Published:** 2017-03-01

**Authors:** Zari P. Dumanian, Viktor Tollemar, Jixing Ye, Minpeng Lu, Yunxiao Zhu, Junyi Liao, Guillermo A. Ameer, Tong-Chuan He, Russell R. Reid

**Affiliations:** 1 Department of Surgery, Section of Plastic Surgery, The University of Chicago Medical Center, Chicago, IL, United States of America; 2 Molecular Oncology Laboratory, Department of Orthopedic Surgery and Rehabilitation Medicine, The University of Chicago Medical Center, Chicago, IL, United States of America; 3 Department of Biomedical Engineering, Northwestern University, Evanston, IL and the Department of Surgery, Feinberg School of Medicine, Northwestern University, Chicago, IL, United States of America; Second University of Naples, ITALY

## Abstract

Large skeletal defects caused by trauma, congenital malformations, and post-oncologic resections of the calvarium present major challenges to the reconstructive surgeon. We previously identified BMP-9 as the most osteogenic BMP in vitro and in vivo. Here we sought to investigate the bone regenerative capacity of murine-derived calvarial mesenchymal progenitor cells (iCALs) transduced by BMP-9 in the context of healing critical-sized calvarial defects. To accomplish this, the transduced cells were delivered to the defect site within a thermoresponsive biodegradable scaffold consisting of poly(polyethylene glycol citrate-co-N-isopropylacrylamide mixed with gelatin (PPCN-g). A total of three treatment arms were evaluated: PPCN-g alone, PPCN-g seeded with iCALs expressing GFP, and PPCN-g seeded with iCALs expressing BMP-9. Defects treated only with PPCN-g scaffold did not statistically change in size when evaluated at eight weeks postoperatively (p = 0.72). Conversely, both animal groups treated with iCALs showed significant reductions in defect size after 12 weeks of follow-up (BMP9-treated: p = 0.0025; GFP-treated: p = 0.0042). However, H&E and trichrome staining revealed more complete osseointegration and mature bone formation only in the BMP9-treated group. These results suggest that BMP9-transduced iCALs seeded in a PPCN-g thermoresponsive scaffold is capable of inducing bone formation in vivo and is an effective means of creating tissue engineered bone for critical sized defects.

## Introduction

There are a variety of treatment options available for the repair of craniofacial defects. While each has distinct advantages, these options are not without their shortcomings. Autografts, generally procured from the cranium, iliac crest, ribs, tibia and other areas are considered to be the gold standard in clinical care due to their osteoconductive and osteoinductive nature [1–2]. However, while this approach epitomizes the clinical dogma of replacing “like with like,” defect size can preclude this option due to the relative limited supply of available autologous tissue. Furthermore, the defect morphology can make autologous reconstruction challenging as these materials can be difficult to contour. Significant donor site morbidity including pain, infection, bleeding, and injury to surrounding critical structures (e.g. pneumothorax in rib harvest) also exist [3–5]. Allografts and alloplasts circumvent supply constraints and provide an alternative for more extensive defects. However, they do so at the added risk of infection, extrusion, and resorption [2]. Finally, biocompatible ceramics, composed of the inorganic aspect of native bone, can be used, but their use is complicated by slow degradation rate, which delays and inhibits ingrowth of newly formed bone [6].

Tissue engineering-based strategies involve three key components: osteoinductive growth factors, osteoprogenitor cells, and biodegradable and osteoconductive scaffolds. Combined, these components facilitate ossification and encourage integration with surrounding bone tissue, even in large critical-sized craniofacial defects that do not fill in with bone over time. In terms of growth factors, the role of BMPs as important bone-forming factors has been well established across many studies [7–9] with BMP2, 6, and 9 demonstrating most significant potential for inducing osteogenic differentiation. Much has been reported regarding BMP2, the most popular and widely used BMP in clinical and experimental medicine [10–13]. Despite the widespread use of BMP2, we previously demonstrated that BMP9 is the most potent osteogenic BMP[14], and as such, may have therapeutic potential for autologous bone growth in the context of adenoviral BMP9 (AdBMP9)-induced cell-mediated osteogenesis. In regards to osteoprogenitor cells, we have isolated and immortalized murine calvarial mesenchymal progenitor cells (iCALs) [15], which have proven to be multipotent and are effectively induced by BMP9 to differentiate into mature bone forming cells in vitro and to produce ectopic bone in vivo in a stem cell implantation assay [16].

Regarding the scaffold component, the ideal scaffold should be able to conform to the shape of the bone defect, easy to handle during surgery, and capable of promoting bone formation at the target site. Poly(polyethylene glycol citrate-co-N-isopropylacrylamide) (PPCN) is a thermoresponsive biomacromolecule with intrinsic antioxidant properties that has been shown to support the entrapment, viability, and function of cells *in vitro* and *in vivo* [17–19]. PPCN can reversibly undergo liquid-to-solid phase change in response to temperature increase from room temperature to 37°C and may be a suitable delivery vehicle for osteogenic growth factors and progenitor cells [20–25]. When mixed with gelatin (0.1%), the resulting interpenetrating network referred to as PPCN-g has been demonstrated to support effective *in vivo* BMP9-induced osteogenesis of stem cells from a variety of sources, including murine embryonic fibroblasts [26] and murine adipocytes [27]. In this study, we investigated whether BMP9-transduced iCALs suspended within PPCN-g would regenerate bone in murine critical-sized calvarial defects.

## Materials and methods

### Recombinant adenoviral vectors

Recombinant adenovirus containing human BMP9 was generated utilizing AdEasy technology, developed by our lab, as previously described [28–30]. Briefly, coding regions taken from BMP9 were amplified via PCR before being cloned into an adenoviral vector. The resultant recombinant adenoviral vector was then used to package and generate the BMP9-expressing adenovirus AdBMP9 in HEK293 cells (American Type Culture Collection, Manassas, VA). In addition to BMP9, the AdBMP9 also expresses green fluorescence protein (GFP), so as to provide an identifiable means for monitoring infection efficiency. Conversely, the control virus was created in a comparable manner that was engineered to express only GFP (AdGFP) [29]. Upon amplification, titrations were completed to determine the optimal viral titers for infection.

### Cell culture and adenoviral infection

**Fig 1:**
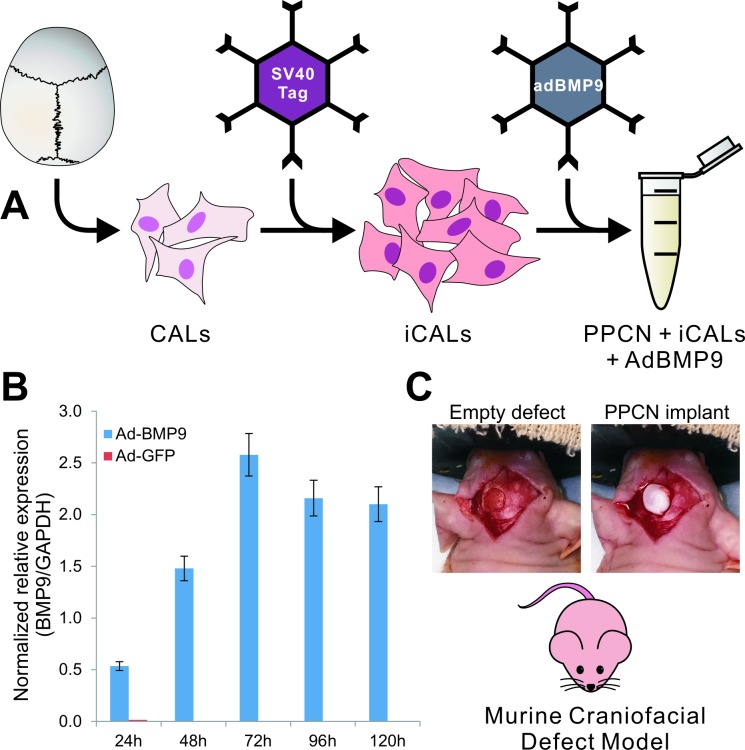
Schematic representation of the overall experimental design. (A) Murine calvarial cells (CALs) were immortalized via retrovirally introduced SV40 large T antigen to produce iCALs. iCALs were then transduced with BMP9 via adenoviral vector and mixed with PPCN scaffolding material. (B) qPCR analysis demonstrating relative expression of BMP9 in iCALs infected with ad-BMP9 (blue bars) compared to control (ad-GFP)-infected cells (red bars). Gene transcript expression was normalized against GAPDH expression. (C) The mixture was subsequently tested in our murine craniofacial defect model. Four millimeter diameter full-thickness calvarial defects were created in the left parietal bone of 8-week-old male athymic (nu/nu) mice. The newly created empty defect reveals the underlying dura mater. PPCN alone or PPCN and adenovirally transduced immortalized calvarial cells were used to fill the defect site.

Mouse calvarial mesenchymal progenitor cells (CALs) have previously been isolated by our lab [15] and have shown the capacity to proceed through osteogenic differentiation in the presence of BMP2 and BMP9 [15, 16]. CALs were reversibly immortalized through retroviral-mediated introduction of the SV40 large T antigen (Fig 1) [29–30]. Immortalized CALs (iCALs) were maintained in complete Dulbecco’s modified Eagle’s medium (DMEM) supplemented with 10% fetal calf serum (FBS; Mediatech, Herndon, Virginia), 1% penicillin, and 1% streptomycin at 37°C in 5% CO_2_. iCALs were passaged to 50% confluence and then infected with AdBMP9 or AdGFP. Polybrene was added in conjunction with virus at a concentration of 2μl/ml of medium to increase infection efficiency [31]. Following 24 hours of exposure to viral vectors, medium was aspirated and cells were trypsinized and resuspended in sterile PBS for mixing with PPCN-g and direct application to the defect sites (see below).

### Cell delivery using PPCN-g

The PPCN was synthesized and characterized as previously described [18, 26]. PPCN was mixed with 0.2% gelatin in a 1:1 ratio in order to improve iCAL adhesion to the scaffold, allow for uniform distribution of iCALs within the scaffold, and increase the viscosity of the PPCN in the liquid phase. iCALs were diluted to a concentration of 1 x 10^6^/μl and 5 μl of cell suspension was mixed with 15 μl of PPCN-g on ice to produce 20 μl aliquots for implantation.

### Establishment of critical murine calvarial defect

All animal surgical procedures were approved by the University of Chicago Animal Care and Use Committee (ACUP # 71745). Eight-week-old male athymic (nu/nu) mice weighing approximately 27 g each were obtained from Harlan Laboratories (Indianapolis, IN). Mice were maintained with general anesthesia (2% isoflurane/100% O_2_) during the procedure and received Meloxicam (1.0mg/kg) subcutaneously perioperatively for analgesia. Seven mice were treated with AdBMP9-transduced iCALs in PPCN-g. Another seven were treated with AdGFP-transduced iCALs in PPCN-g, serving as controls. Four mice were treated with PPCN-g alone and served as the scaffold control. Our pilot experiments and prior experience with the model have shown that an untreated empty defect fails to close, and thus this group was omitted.

Under sterile conditions, a sagittal incision was made from the orbital ridge to 5 mm behind the ears. Skin flaps on either side were retracted to expose the calvaria. A Dremel (Racine, WI) MultiPro cordless handheld drill fitted with a stainless steel trephine drill bit was used at low speed to create a full-thickness 4mm-diameter calvarial defect on the left parietal bone of each mouse. Sterile phosphate buffered saline (PBS) with 1000 IU/ml penicillin and 1 mg/ml streptomycin (35 ml/kg) was used for irrigation during drilling. 20 μl of implant material with or without adenoviral infected iCALs was instilled into the defects using a P200 pipet and left in place to solidify for approximately five minutes. Importantly, all implant aliquots were kept on ice until just prior to injection in order to both improve ex vivo iCAL viability and to prevent premature solidification during implantation. Incisions were closed using 5–0 nylon interrupted sutures, which were removed after 10–14 days. The animals were monitored after anesthesia hourly in the immediate postoperative period. If pain medications were needed, they were administered only after the mice have recovered and it was safe to administer these medications. Pain medication was given as needed and if evidence of pain (i.e., hunched posture, ruffled fur) was observed. Post-procedural analgesics used were buprenorphine 50ug/kg q 12 hours for the first 48 hours and Meloxicam 1mg/kg q 24 hrs PRN. Thereafter, the subjects were inspected on a daily basis for signs of distress, or changes in CNS function. Any mouse that developed signs of post-surgical stress, or was not moving when stimulated was euthanized. The mice were weighed daily for the first three days then 2–3 times each week for the first two weeks post-surgery. All mice were housed in a standard barrier facility, exposed to temperature-controlled conditions with 12 h light/dark cycles and fed a normal diet at the University of Chicago Animal Resource Center (ARC). A schematic and photographic representation of our methodology, including cell immortalization and adenoviral infection, murine critical-sized defect creation, and cell delivery using PPCN-g scaffold are provided (Fig 1). Adenoviral infection of experimental iCALs using Ad-BMP9 demonstrated significant target gene expression out to 120 hours post-infection compared to control (Ad-GFP) infected cells (Fig 1B).

### MicroCT imaging and data analysis

To monitor the progression of osteogenesis, mice underwent serial microCT imaging at 48 hours, 2, 4, 6, 8, and 12 weeks postoperatively. During scanning, the mice were sedated using the same isoflurane delivery procedure described previously [32, 33]. Mice were scanned on a trimodality Triumph (Trifoil, Northridge, CA), with the following parameters: voltage: 60kV current: 140 mA; field of view: 33.80 mm; projections: 512; pixel size: 66 um. The images were analyzed with Amira® 5.0 software (FEI, Hillsboro OR). Scale bars were used to standardize the images.

Baseline imaging and defect volume calculations (using volumetric reconstructions generated in Amira®) were performed at 48 hours postoperatively and served as a standard to which all subsequent residual defect volume measurements were compared. Bone regeneration volume was scored using one-way ANOVA with a significance cutoff of p ≤ 0.05 (S1 Table). Two-tailed t-tests were used to assess defect size relative to baseline (week 0) at various time intervals, as well as to compare percentage of defect filled between AdBMP9 treated and AdGFP treated groups (S2 Table).

### Histological analysis

Following the last microCT imaging, the mice were euthanized with CO_2_ inhalation and subsequent cervical dislocation. The skull samples containing defect sites were retrieved, fixed in formalin, decalcified, and paraffin-embedded, followed by sectioning and subjecting to H & E staining and trichrome staining as previously described [30].

## Results and discussion

### BMP9-transduced iCALs delivered by PPCNg scaffold can effectively repair cranial defect

In terms of morbidity and mortality, all animals survived the surgery. However, two out of seven mice in the iCAL-BMP9 treatment group died within 24 hours of surgery and one of seven mice in the iCAL-GFP control group died 11 weeks postoperatively. Other complications were technical in nature. During the procedure of a mouse from the iCAL-GFP group, the implant was inadvertently displaced from the defect site prior to solidification and could not be recovered. CT imaging data from an additional mouse in the iCAL-BMP9 group at week 2 showed bone formation in a pattern that suggested lateral displacement of the implant. Data from all of the above mentioned mice were omitted from analysis. All surviving mice progressed well postoperatively, gaining an average 1.2 grams/week and apparently with well-treated pain.

**Fig 2:**
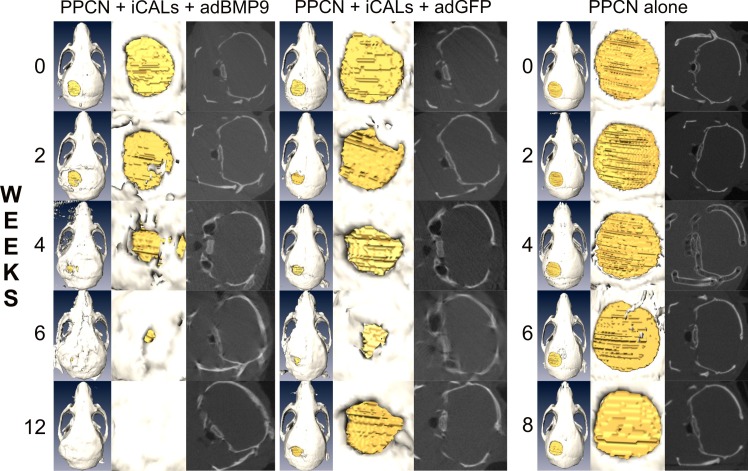
Time-course microCT imaging of the calvarial defects. At 24–48 hours postoperatively, baseline microCT imaging was performed and analyzed to determine defect volume. Follow-up imaging and analysis was performed at 2, 4, 6, 8, and 12 weeks postoperatively to quantify residual defect volume and new bone ossification. Representative images are shown.

**Fig 3:**
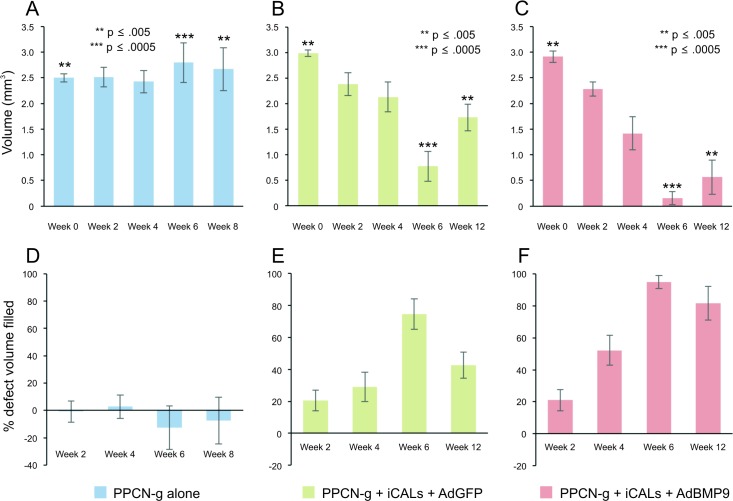
Quantitative analysis of the BMP9-induced calvarial defect repair. (A, B, C) Average defect volumes (mm^3^) were calculated at 24–48 hours, 2, 4, 6, 8, and 12 weeks postoperatively using volumetric reconstructions generated in Amira®. Asterisks indicate a significant (p < 0.05) difference in average defect volume between test groups at the specified time point, as determined by one-way ANOVA. (D, E, F) The change in defect volume over time was used to deduce the percentage of baseline defect volume filled with new bone.

In order to assess the dynamic process of osteogenesis at the repair sites, microCT imaging was performed at 48 hours, 2, 6, 7, 8, and 12 weeks postoperatively (Fig 2). Volumetric reconstructions were generated using Amira® and defect volumes were quantitatively determined. Baseline imaging at 48 hours postoperatively revealed uniform circular defects, with average volumes of 2.99 mm^3^, 3.00 mm^3^, and 2.50 mm^3^ for PPCN-g/AdBMP9 (Fig 3C), PPCN-g/AdGFP (Fig 3B), and PPCN-g alone (Fig 3A), respectively. A significant difference between baseline defect volumes of our test groups was detected via one-way ANOVA (p = 0.0049).

Despite starting with the statistically smallest defects, the bone gaps treated only with PPCN-g scaffold did not statistically change in size when evaluated eight weeks postoperatively (p = 0.72). Conversely, both animal groups treated with iCALs showed significant reductions in defect size compared to baseline (week 0) after 12 weeks of follow-up (AdBMP9-treated: p = 0.0025; AdGFP-treated: p = 0.0042). A significant difference between test groups was detected by one-way ANOVA by 6 weeks of follow-up (p = 0.0003) (Fig 3A–3C). The PPCN-g/AdBMP9-treated defects failed to exhibit greater bone regeneration compared to the control (PPCN-GFP) group at 6 weeks postoperatively (p = 0.11); however, by 12 weeks, significantly greater bone regeneration was apparent in the BMP9 treatment group. (p = 0.027) (Fig 3E and 3F). Interestingly, between 6 and 12 weeks, both iCAL-treated groups showed an increase in average defect size, the extent of which was more than twice as great in the PPCN-g/AdGFP-treated group (0.96 mm^3^ increase) compared with the PPCN-g/AdBMP9-treated group (0.41 mm^3^ increase). This may be attributed to better bone formation in the BMP group than in the GFP group although exact causes require further investigation.

### BMP9 promotes complete osteointegration and mature bone formation at the cranial defect sites

**Fig 4:**
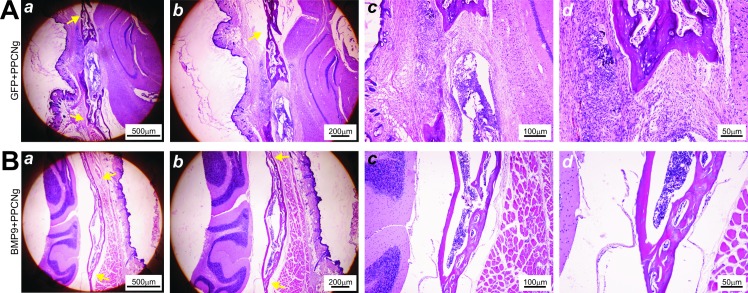
Histologic evaluation of the BMP9-induced calvarial defect repair. Histologic analysis of tissue microsections harvested 12 weeks post-treatment. Yellow arrows indicate the original defect borders. (A) The defect site of this AdGFP-treated mouse shows incomplete healing; there is some ingrowth of bone, but fibrous tissue fills part of the defect. (B) Conversely, the defect site of an AdBMP9-treated mouse has been completely bridged with new bone. All sections show no trace of PPCN material, indicating complete resorption.

**Fig 5:**
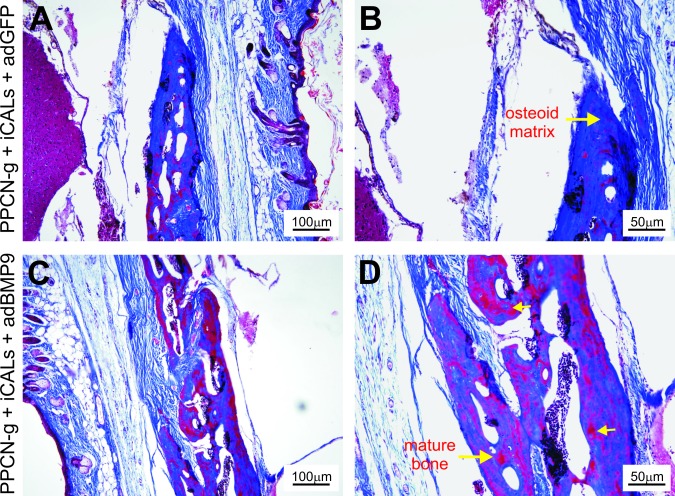
Assessment of the Trichrome histology. Histologic analysis of tissue microsections harvested 12 weeks post-treatment. (A, B) Chondroid matrix and a smaller proportion of mature bone is shown in the defect site of a PPCN + iCAL + AdGFP-treated mouse. (C, D) A visibly higher proportion of mature bone can be appreciated in samples taken from the defect sites treated with PPCN + iCAL + AdBMP9.

While microCT imaging provides an assessment of the dynamics of the defect repair process, the quality of bony repair at defect sites requires examination by histologic analysis. H & E and trichrome staining of the retrieved samples confirmed incomplete healing in PPCN-g/AdGFP-treated group as the defects were primarily bridged with fibrous tissue (Fig 4A) and cartilage (Fig 5A and 5B). Conversely, robust bone formation was seen in the PPCN-g/AdBMP9-treated group, with some defects filled in completely with new bone (Fig 4B). Trichrome staining of calvarial defect microsections harvested at 12 weeks post-treatment corroborated these findings by showing a higher proportion of mature bone in the PPCN-g/AbBMP9-treated defect sites (Fig 5C and 5D) than in those treated with PPCN-g/AdGFP (Fig 5A and 5B). In all samples, no trace of PPCN-g material was observed, suggesting that the scaffold was completely degraded, which was further confirmed in the PPCN-g alone group (S1 Fig).

### Combining BMP9 induction and cell delivery via the PPCN-g scaffold is a promising stem cell-based therapeutic strategy for cranial defect repair

Efficacious bone regeneration requires integration with surrounding tissue, including vascularization, fusion of the implant with autologous bone without fibrous tissue at the bone-implant interface, and eventual complete replacement of the scaffold with new bone [34–36]. In this study we have demonstrated that a novel class of thermoresponsive biomaterial loaded with BMP9-induced calvarial cells is capable of producing bone that successfully integrates with neighboring tissue.

One of the components contributing to this demonstration was the PPCN-g scaffold, which provided a temporary framework for osteoblastic differentiation and osteogenesis. Histology confirms bone formation, and complete degradation of the scaffold. We did not obtain and analyze serial histological samples and therefore cannot thoroughly comment on the degradation kinetics of PPCN-g, but previous *in vitro* studies have determined that the material undergoes hydrolytic breakdown over a period of several weeks and is resorbed by the body [23]. Grossly, no signs of infection, dehiscence, or scaffold extrusion were observed.

Another component contributing to successful bone regeneration and defect healing was BMP9 and its ability to induce osteoblastic differentiation of iCALs. BMP9 has previously exhibited considerable potency in osteoblastic induction [14], and utilization of adenoviral vector gene therapy permits localization of BMP-secreting cells [15,16]. Trichrome staining of histologic microsections harvested at 12 weeks postoperatively demonstrated bridging of those defects with fibrocartilage in the GFP only treatment group rather than bone in the BMP-9 treatment group. Both microCT and histology results support bone formation only in the PPCN-g + iCALs + AdBMP9-treated defects. BMP9 appears to induce direct osteogenic differentiation, leading to intramembranous ossification without a fibrocartilage intermediate. Interestingly, without BMP-9 stimulation, the Ad-GFP infected iCALs embedded in PPCN-g appear to assume a cartilaginous phenotype upon differentiation (Fig 5A and 5B). This chondroid phenotype is a consistent finding no matter what progenitor cell source we have used in our experimentation—iCALs [15,16], immortalized murine embryonic fibroblasts (iMEFs) [26], immortalized murine adipocytes (iMADs) [27], and may represent a differentiation “default” for cells without BMP9 stimulation in the PPCN microenvironment. Overall, these results reiterate previous findings from our lab that demonstrate the superior ectopic bone quality produced by BMP9-transduced iCALs compared to that produced by GFP-transduced iCALs [15,16].

The novelty of this study is threefold. First, this is the first study to demonstrate Ad-BMP-9 induced osteogenesis is effective in the repair of a critical-sized calvarial defect. Secondly, we have shown that immortalized calvarial progenitor cells can be induced in vivo to repair osseous defects of the skull in the mouse. Thirdly, we have introduced a recently developed thermoresponsive material, PPCN-g in the context of craniofacial reconstruction. The decision to use PPCN-g was made based on two major advantages over traditional solid scaffolds. First, because of its initial liquid phase that subsequently solidifies upon introduction into the defect, PPCN-g spontaneously molds to the shape of even the most complicated defects, which may be beneficial with the complex three-dimensional morphology of the skull and facial bones. Second, liquid-phase PPCN-g has the potential to be delivered through minimally invasive means. This has important implications for reducing scaffold dislodgement and contour irregularities that can arise from imprecise scaffold fit. An additional benefit may be the addition of gelatin to PPCN, which provides cell adhesion sites for osteoconduction. Recent data from our laboratory suggest that the addition of gelatin (0.1%) to PPCN enhances angiogenesis within the material. [26]. Specifically, PPCN-g seeded with BMP9-transduced immortalized mouse embryonic fibroblasts (iMEFs) produced statistically more mature bone and VEGF expression than similarly transduced iMEFs in an *in vivo* ectopic bone assay [25]. Such an interpenetrating network, in combination with BMP9, may be an effective strategy to produced well-vascularized, mature bone in defects under even more hostile conditions such as radiation or infection.

Although the results presented herein are promising, there are limitations to the study. A major limitation of this study is the small sample size of the three groups. Secondly, there were minor differences in initial cranial defect size, however it is important to note that the treatment group defect size was slightly larger than the control group, which re-emphasizes the potentially important therapeutic role of BMP-9 in critical-sized cranial defect repair. Some may argue that another limitation of this study and its digression from clinical relevance is the use of adenovirus to express BMP-9 in infected progenitor cells. To this end, recent studies have investigated the efficacy of recombinant human BMP-9 in osteogenic differentiation and cranial defect repair [37, 38]. Although these studies reiterate our finding of the superior potency of BMP-9 over other osteogenic BMPs [37], and the ability to regenerate new bone in rat critical size calvarial defects [38], several issues subvert the use of rhBMP9. First, the recombinant form is highly expensive, as it is purified from CHO or HEK 293 cells. Furthermore, to date there is no recombinant form yet to demonstrate osteoactivity from bacterial sources. Therefore, applications to repair large cranial defects in humans, the sole driving force to investigate tissue engineering strategies, using rhBMP9 will be confounded by its prohibitive cost. Additionally, as the biological activity of this recombinant protein is largely unknown, achieving the correct dose-response relationship will be challenging to align bioavailability of the protein with healing time needed for complete reconstruction of a given defect. Along these lines, the representative histology provided in the literature regarding its use in critical sized calvarial defects demonstrate incomplete healing of the treated defects, with chondroid and osseous material present in the regenerate [38].

Despite achieving statistically significant differences in defect filling between all three groups (CI 95%), future studies should include larger sample sizes. Lastly, we propose that subsequent iterations of this study be expanded and scaled to utilize larger animals such as rabbits or pigs.

## Conclusions

We conclude that AdBMP9-transduced iCALs seeded in a PPCN-g scaffold is capable of inducing bone formation in vivo and is an effective means of craniofacial defect repair in mice. This is supported by microCT evidence of defect filling and histological confirmation of mature bone regeneration. **The combination of AdBMP-9 progenitor cell therapy and a PPCN-g microenvironment** represents a promising and innovative approach to craniofacial defect repair.

## Supporting information

S1 Table One-way ANOVA analysis of bone regeneration volume. (PDF)

S2 Table Raw data of defect volume size (mm3) for PPCN-g alone, Ad-GFP and Ad-BMP9 treated groups. Averages (AVG), standard deviation (SD) and standard error of the mean (SE) for each group and time point listed.
(XLSX)

S1 Fig Histological analysis of calvarial defects harvested from mice treated with the PPCNg scaffold alone. H&E (panel A) and trichrome (panel B) staining of microsections obtained from calvarial specimens harvested at 8 weeks post-craniotomy and treatment demonstrate mainly fibrotic tissue within the defects.
(TIF)
