# Supplementary material for: Repair of critical sized cranial defects with BMP9-transduced calvarial cells delivered in a thermoresponsive scaffold
Source: PLoS One. 2017 Mar 1;12(3):e0172327. doi: 10.1371/journal.pone.0172327 (PMC5332017; doi:10.1371/journal.pone.0172327)
Supplement: S1 Table — (PDF) [file pone.0172327.s001.pdf]

	1way ANOVA					
1	Table Analyzed	Week 0				
2						
3	One-way analysis of variance					
4	P value	0.0049				
5	P value summary	**				
6	Are means signif. different? (P < 0.05)	Yes				
7	Number of groups	3				
8	F	9.490				
9	R square	0.6549				
10						
11	ANOVA Table	SS	df	MS		
12	Treatment (between columns)	0.5868	2	0.2934		
13	Residual (within columns)	0.3092	10	0.03092		
14	Total	0.8959	12			
15						
16	Bonferroni's Multiple Comparison Test	Mean Diff.	t	Significant? P < 0.05?	Summary	95% CI of diff
17	PPCN vs BMP9	-0.4156	3.342	Yes	*	-0.7724 to -0.05873
18	PPCN vs GFP	-0.4879	4.136	Yes	**	-0.8264 to -0.1493
19	BMP9 vs GFP	-0.07230	0.6130	No	ns	-0.4108 to 0.2662

1way ANOVA

1	Table Analyzed	Week 2				
2						
3	One-way analysis of variance					
4	P value	0.7389				
5	P value summary	ns				
6	Are means signif. different? (P < 0.05)	No				
7	Number of groups	3				
8	F	0.3120				
9	R square	0.05873				
10						
11	ANOVA Table	SS	df	MS		
12	Treatment (between columns)	0.1045	2	0.05227		
13	Residual (within columns)	1.675	10	0.1675		
14	Total	1.780	12			
15						
16	Bonferroni's Multiple Comparison Test	Mean Diff.	t	Significant? P < 0.05?	Summary	95% CI of diff
17	PPCN vs BMP9	0.2278	0.7869	No	ns	-0.6029 to 1.058
18	PPCN vs GFP	0.1300	0.4733	No	ns	-0.6581 to 0.9180
19	BMP9 vs GFP	-0.09780	0.3562	No	ns	-0.8859 to 0.6902

	1way ANOVA					
1	Table Analyzed	Week 4				
2						
3	One-way analysis of variance					
4	P value	0.0906				
5	P value summary	ns				
6	Are means signif. different? (P < 0.05)	No				
7	Number of groups	3				
8	F	3.083				
9	R square	0.3814				
10						
11	ANOVA Table	SS	df	MS		
12	Treatment (between columns)	2.163	2	1.082		
13	Residual (within columns)	3.508	10	0.3508		
14	Total	5.671	12			
15						
16	Bonferroni's Multiple Comparison Test	Mean Diff.	t	Significant? P < 0.05?	Summary	95% CI of diff
17	PPCN vs BMP9	1.007	2.404	No	ns	-0.1952 to 2.209
18	PPCN vs GFP	0.2937	0.7393	No	ns	-0.8467 to 1.434
19	BMP9 vs GFP	-0.7132	1.795	No	ns	-1.854 to 0.4272

	1way ANOVA					
1	Table Analyzed	Week 6				
2						
3	One-way analysis of variance					
4	P value	0.0003				
5	P value summary	***				
6	Are means signif. different? (P < 0.05)	Yes				
7	Number of groups	3				
8	F	21.01				
9	R square	0.8078				
10						
11	ANOVA Table	SS	df	MS		
12	Treatment (between columns)	15.45	2	7.725		
13	Residual (within columns)	3.676	10	0.3676		
14	Total	19.13	12			
15						
16	Bonferroni's Multiple Comparison Test	Mean Diff.	t	Significant? P < 0.05?	Summary	95% CI of diff
17	PPCN vs BMP	2.640	6.158	Yes	***	1.410 to 3.871
18	PPCN vs GFP	2.020	4.967	Yes	**	0.8530 to 3.188
19	BMP vs GFP	-0.6199	1.524	No	ns	-1.787 to 0.5475

1way ANOVA							
1	Table Analyzed	Week (8)12					
2							
3	One-way analysis of variance						
4	P value	0.0051					
5	P value summary	**					
6	Are means signif. different? (P < 0.05)	Yes					
7	Number of groups	3					
8	F	9.369					
9	R square	0.6520					
10							
11	ANOVA Table	SS	df	MS			
12	Treatment (between columns)	8.922	2	4.461			
13	Residual (within columns)	4.761	10	0.4761			
14	Total	13.68	12				
15							
16	Bonferroni's Multiple Comparison Test	Mean Diff.	t	Significant? P < 0.05?	Summary	95% CI of diff	
17	PPCN vs BMP9	2.107	4.319	Yes	**	0.7069 to 3.508	
18	PPCN vs GFP	0.9388	2.028	No	ns	-0.3897 to 2.267	
19	BMP9 vs GFP	-1.168	2.524	No	ns	-2.497 to 0.1600	
