# Supplementary figures and images for: Repair of critical sized cranial defects with BMP9-transduced calvarial cells delivered in a thermoresponsive scaffold
Source: PLoS One. 2017 Mar 1;12(3):e0172327. doi: 10.1371/journal.pone.0172327 (PMC5332017; doi:10.1371/journal.pone.0172327)

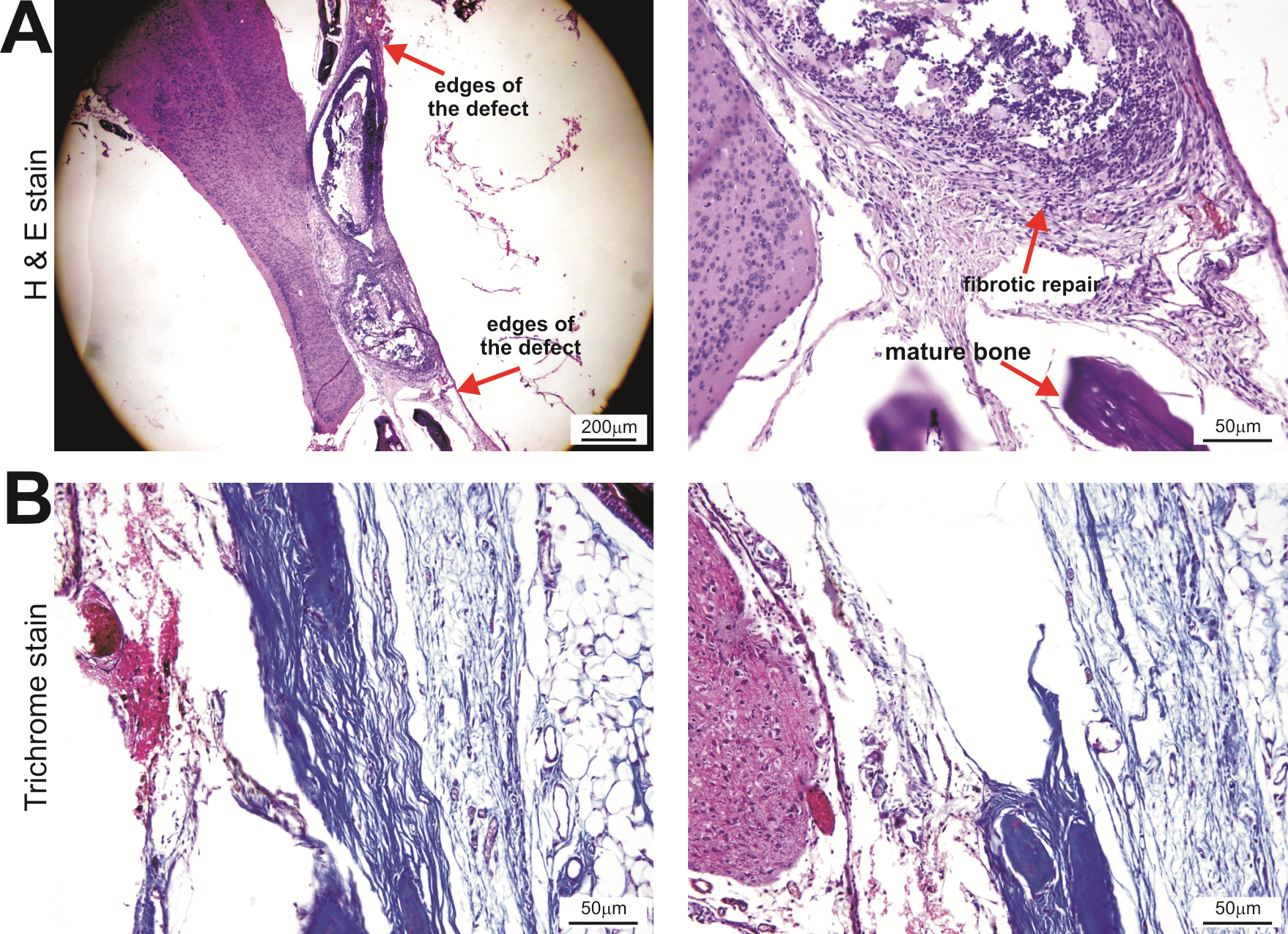

Supplement: S1 Fig — H&E (panel A) and trichrome (panel B) staining of microsections obtained from calvarial specimens harvested at 8 weeks post-craniotomy and treatment demonstrate mainly fibrotic tissue within the defects. (TIF) [file pone.0172327.s003.tif]
